# Hemispherical Cell-Inspired Soft Actuator

**DOI:** 10.3389/fbioe.2020.00020

**Published:** 2020-02-19

**Authors:** Kahye Song, Youngsu Cha

**Affiliations:** ^1^Center for Intelligent & Interactive Robotics, Korea Institute of Science and Technology, Seoul, South Korea; ^2^John A. Paulson School of Engineering and Applied Sciences, Harvard University, Cambridge, MA, United States; ^3^Wyss Institute for Biologically Inspired Engineering, Harvard University, Cambridge, MA, United States

**Keywords:** soft actuator, bio-mimicry, sensor embedded actuator, electrostatic actuator, layer actuator

## Abstract

As soft robots have been popular, interest in soft actuators is also increasing. In particular, new types of actuators have been proposed through biomimetics. An actuator that we proposed in this study was inspired by a motor cell that enables plants to move. This actuator is an electrostatic actuator utilizing electrostatic attraction and elastic force, and can be used repeatedly. In addition, this actuator, which can produce large and diverse movements by collecting individual movements like a cell, has a wide application field. As one of them, this actuator is stacked to construct a layer structure and propose an application example. In addition, a piezo sensor was built inside the actuator and real-time motion monitoring was attempted. As a result, the point laser sensor value and the piezo sensor value coincided with each other, which means that it is possible to detect motion in real-time with the built-in sensor.

## Introduction

Soft robots are an indispensable device to be used in various fields. Soft robots can use dexterous mobility by using mechanisms different from conventional hard robots and it guarantees great potential beyond previous limitations (Trivedi et al., 2008; Laschi and Cianchetti, 2014; Martinez et al., 2014; Rus and Tolley, 2015). This allows the robot tip to reach any point in the 3D workspace with an infinite robot shape or configuration, and is less resistant to compressive forces and can carry fragile objects without damaging it. The soft robots are therefore ideal for various applications such as personal robots interacting with humans, service robots to reach confined spaces, medical robots especially used in surgery, and defense and structural robots operating in unsteady environments (Trivedi et al., 2008; Majidi, 2014).

Biomimetics has received huge attention as a new idea and solution for soft robotics development (Cho et al., 2009; Majidi, 2014; Rus and Tolley, 2015). By studying how animals move in complex, unpredictable environments using soft materials, we can gain valuable insight into emerging robot applications in the areas of medicine, search and rescue, disaster response, and human resource support (Kim et al., 2013). Thus, the soft robots have already been obtained through a biomimetic technique and have been introduced (Majidi, 2014). A soft manipulator was made using the muscle characteristics of the octopus and a robot capable of ballistic rolling motion was made by simulating larvae (Lin et al., 2011; Laschi et al., 2012). Swimming robot, earthworm robots, and jellyfish robot were developed by mimicking locomotion of microorganisms and mollusks (Quillin, 1998; Yeom and Oh, 2009; Palagi et al., 2016; Shintake et al., 2018; Wen et al., 2018). For these smooth motions, soft actuators are essential. Thus, with the development of soft robots, various types of soft actuators are being developed to realize biomimetic technology (Jung et al., 2007; Trimmer et al., 2012; Marchese et al., 2014; Gu et al., 2017). In addition, inspired by nature including starfish and octopus’s arm, the new types of soft actuators were developed (Trivedi et al., 2008; Laschi et al., 2009, 2012; Yoshida, 2010).

**FIGURE 1:**
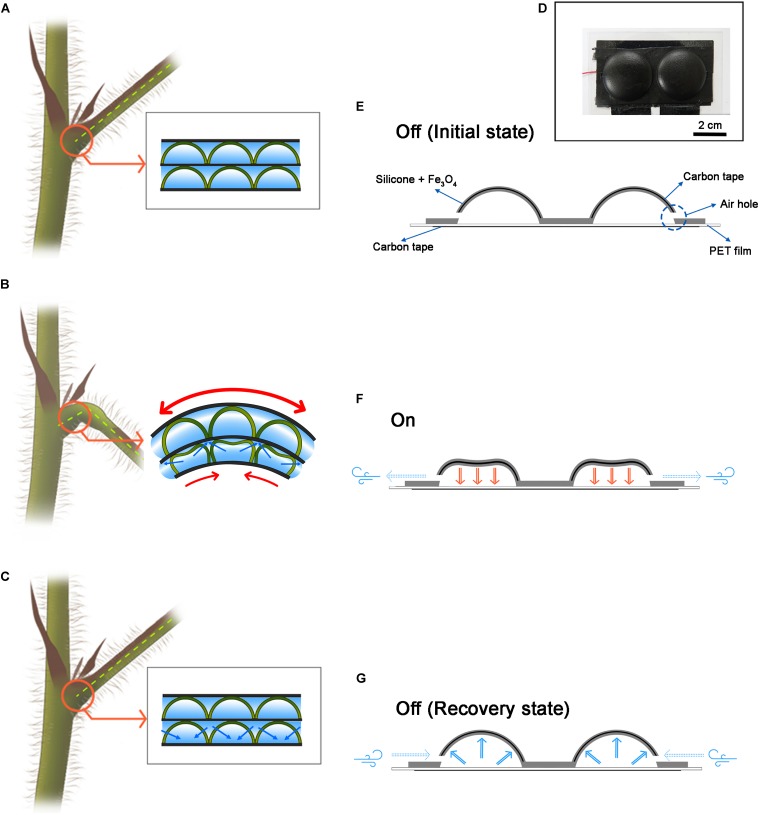
Motor cell-inspired actuator and schematic for operation. **(A)** The motor cell schematic inside the plant. **(B)** When the water inside the swelled motor cell disappears and the force to sustain is lost, the branches fall down. **(C)** When water enters the motor cell, the cell expands and recovers to its original state. **(D)** An actuator inspired by a motor cell. This actuator was fabricated in 1 × 2 structure. **(E)** Initial state of the CI actuator. It also describes the components of the CI actuator. **(F)** When the CI actuator is on state. The electrostatic attraction forces to the floor and the air escapes through the air hole. **(G)** When the CI actuator is in the off state. It is restored to its original shape by elasticity, and air is flowed from the outside.

So far, most biomimetics techniques for soft robots have been mainly focused on animals such as mollusks, marine animals, and insects, but one of the greatest inspirational objects is plants (Bar-Cohen, 2006; Dicker et al., 2014; Zhang et al., 2016). Venus flytrap and the pitcher spatula catches worms, and the pine cones close the shell for seed protection (Dumais and Forterre, 2012). Although the movement of various plants is interesting, a representative plant is *Mimosa pudica*. Mimosa responds to external stimuli by rapidly folding the leaves and dropping the stem down. It is the most rapid hydraulic motion among the multicellular movements in plants and fungi (Skotheim and Mahadevan, 2005). In addition, the moving direction is not simply one direction but an omnidirectional motion (Song et al., 2014). It is the motor cell that enables this fast and wide range of motion (Fleurat-Lessard et al., 1997; Dumais and Forterre, 2012). Motor cells act like hinges on the joints, enabling the movement of plant parts, such as closing and opening leaflets for light intensity and rotating branch in pulvinus. Increasing or decreasing the pressure of the motor cells plays a crucial role in mimosa movement, which can be observed by NMR imaging (Tamiya et al., 1988). When the water disappears in the motor cells supporting the lower part of the branch (Figure 1A), it no longer supports the branch and falls down (Figure 1B). When the water enters the motor cell again, the cell pressure returns to its original shape (Figure 1C). This mechanism is simple and clear, but it allows a very large range of motion. Thus, inspired by these motor cells, we have developed a cell-inspired (CI) actuator (Figure 1D).

Conventional robots are mainly designed to be rigid, allowing them to perform fast, accurate, powerful, and repetitive position control tasks (Kim et al., 2013; Laschi and Cianchetti, 2014; Martinez et al., 2014). Common robots in these robotic systems consist of rigid electromagnetic components (e.g., magnets, copper, and steel bearings) or internal combustion engines made of steel and aluminum alloys. In contrast, soft material takes precedence in the bio-inspired technique (Ilievski et al., 2011; Kim et al., 2013; Rus and Tolley, 2015). Most creatures are made up of soft tissues and liquids. Thus, one of the biggest challenges of soft robots is to design a flexible working system with flexible materials that can exert high power to reproduce the muscle function of the creatures.

A typical technique is the use of dielectric elastomer actuators (DEAs) made of a soft material that works through electrostatic forces (Kim et al., 2013; Gu et al., 2017). The field-induced activation reaction of the DEA is caused by the electrostatic attraction between the two electrically conductive layers applied to the surface of the polymer membrane (Shankar et al., 2007). The voltage potential difference is applied between the two compliant electrodes to compress the thickness and elongation of the polymer region. On the other hand, ion electroactive polymer (EAP) actuators operate by migration of mobile ions within the polymer (Bar-Cohen et al., 2001; Bar-Cohen, 2002; Chen and Zhu, 2015). Small changes in the external variables such as electric and magnetic fields, temperature, solvent quality, and pH result in discontinuous changes. Examples of ionic EAPs include polymer electrolyte gels, ionic polymer–metal composites (IPMCs), conductive polymers, and bucky gel actuators (Chen and Zhu, 2015). In particular, elastic actuators based on silicone materials and elastomer electrodes have many advantages in thickness and manufacturing (Li et al., 2018). In addition, when the elasticity of the silicone material is utilized, it is quickly recovered to its original shape when the actuator is turned off.

One of the important technologies for operating multiple actuators is self-sensing technology. Most soft actuators interfere with effective sensing and control functions due to lack of real-time sensory feedback (Amjadi and Sitti, 2018). Self-sensing technology provides important information to the user by self-measuring the actuator’s operation at the same time (Rakotondrabe et al., 2014). Various self-sensing methods based on dielectric elastomer (DE) actuators/sensors have been successfully developed and evaluated to extract accurate displacement information during the operating process (Jung et al., 2008; Rosset et al., 2013; Rizzello et al., 2018). Self-sensing algorithms have also been developed by using arbitrary voltage oscillations in the sense of DEA capacitance during operation in a manner that can withstand significant changes in electrode resistance and leakage current (Gisby et al., 2013). Also, self-sensing ionically conductive polymeric metal composite actuators have also been developed by providing self-positioning feedback (Punning et al., 2007). Discrete electrothermal stimulation and deformation sensing are also achieved by an optimal combination of graphite microparticles in the form of hybrid films and carbon nanotubes (CNTs) (Amjadi and Sitti, 2018). Lastly, a typical method is to use a piezoelectric sensor (Dosch et al., 1992; Rakotondrabe et al., 2014; He et al., 2018). Truly collocated sensor and actuator measure the position change of the actuator. This self-sensing technique allows simultaneous sensing of displacement and force to implement an interactive control strategy that eliminates the need for additional electromechanical transducers.

Therefore, we fabricated the CI actuator as a mixture of Fe_3_O_4_ and silicone and designed to operate with electrostatic force. At temperatures above 120 K, Fe_3_O_4_ is a good electrical conductor with high conductivity compared to Fe_2_O_3_ (Greenwood and Earnshaw, 1997). Thus, this mixture exhibits flexibility as well as dielectric properties and can also respond to magnetism (Song and Cha, 2018). In a dielectric polymer, the dipoles can be randomly oriented, but the application of a high electric field results in dielectric behavior by aligning the dipoles (Lovinger, 1983; Chen and Zhu, 2015). The rotating spherical iron oxide particles contain dipoles and the dielectric polarization direction is parallel to the external electric field (Song and Cha, 2018). Fe_3_O_4_ is very cheap, has high accessibility, and has high conductivity, but it can be replaced by various conductive particles other than Fe_3_O_4_. In addition, the CI actuator is a pneumatic soft actuator and can be manufactured in various shapes and sizes. Also, because it is made by simulating cells, it is possible to move individual cells. In this study, we will show the structure composed of lamination method among various utilization methods and also the sensor integrated structure will be introduced.

## Materials and Methods

### Making Mold for CI Actuator

The mold was designed using SolidWorks software (Dassault Systèmes SolidWorks Corp., United States) to create the CI actuators with constructible convex and concave parts. Each mold had various sizes by controlling the height and radius. Notably, there was the gap between two convex and concave molds of 0.5 mm so that the CI actuator fabricated by the molds has a thickness of 0.5 mm. This design was printed using a 3D printer (ProJet HD 3500, 3D Systems Inc., United States) [Materials: part (VisiJet M3 Crystal, 3D Systems Inc., United States) and supporter (VisiJet S300, 3D Systems Inc., United States)]. In order to remove the supporter of the output mold, it was placed in a 75°C convection oven (DCF-31-N, Dae Heung Science, South Korea) for 6 h. All remaining supporters were removed from the oil bath in an ultrasonic cleaner (SaeHan Ultrasonic Co., South Korea). After washing and drying, in order to prevent silicone from sticking to the surface of the mold, a release agent (Ease release 200, Smooth-On, Inc., United States) was sprayed and dried for 30 min.

### Fabrication Method for CI Actuator

First, Ecoflex 0030 part A (Smooth-On, Inc., United States), Ecoflex 0030 part B (Smooth-On, Inc., United States), and a curing agent accelerator (Plat-Cat, Smooth-On, Inc., United States) were mixed at a ratio of 1:1:0.04. Extra pure tri-iron tetra-oxide powder (Daejung Chemical & Metal Co., Ltd., South Korea) was mixed with uncured silicone gel at 5% of total volume. The bubbles were removed using a vacuum pump, and the mixture was poured into the concave mold. Carbon conductive double-faced adhesive tape (Nisshin EM Co., Ltd., Japan) to be cut at a width of 15 mm was placed on the mixture. The carbon conductive double-sided adhesive tape is an electrode for supplying power to the mixture from an external power supply device. Then, an additional mixture layer was formed by one more pouring. The convex mold was installed on the concave mold, and the whole molds were fixed with clamps to seal the gap. After about 6 h at room temperature, the clamps were removed and the cured CI actuator was carefully removed using tweezers.

Another carbon conductive double-faced adhesive tape was cut to the same size as the surface area of the CI actuator. The carbon tape was used for a bottom electrode. A 100-μm-thick polyethylene terephthalate (PET) film (Saehan, South Korea) was utilized to prevent short circuit by direct contact of the CI actuator and the carbon tape. The PET was cut with a margin of 5 mm on each of the four sides. Before the insertion, the PET film underwent a flattening process by placing it between heavy glass plates in an oven at 85°C for 6 h.

### Making a Sensor Embedded CI Actuator

Piezo film (28 μm PVDF Silver Ink, Measurement Specialties, Inc., United States) was cut to a size of 30 mm (length) × 5 mm (width) for use as a sensor. A 2 mm × 5 mm copper tape (1181, 3M, United States) was attached to the front and back sides of the sensor and soldered to 0.7-mm-diameter wires.

Similarly, Ecoflex 0030 part A (Smooth-On, Inc., United States), Ecoflex 0030 part B (Smooth-On, Inc., United States), and a curing agent accelerator (Plat-Cat, Smooth-On, Inc., United States) were mixed at a ratio of 1:1:0.04 for pure silicone gel fabrication.

On the CI actuator, silicone gel mixture was deposited thinly. The sensor was placed on top of it for electric isolation, thinly coated with silicone gel, and then hardened at room temperature for about 1 h. The sensor output was measured by the Arduino board (UNO, Arduino, Italy), and the sensor values and time data were transferred to a computer via Bluetooth. Herein, a 30-Hz low-pass filter was used to attenuate the 60-Hz noise from power supply.

### Circuit Configuration and Setup

A high-voltage converter (AG 50P-5, XP Power, Singapore) to output 5 kV was utilized to operate the CI actuator. The high-voltage converter was supplied by a power supply (MK3003P, MK power, South Korea), and its control pin was connected to a waveform generator (33500B Series, Keysight Technologies, United States), which can output square waves (Supplementary Figure 1). Then, a thick film resistor (50M ohms, Ohmite, United States) was connected between the output pins of this converter for discharging.

The (+) and (−) ports of the high-voltage converter were connected to the bottom carbon tape and the CI actuator, respectively.

### Motion Tracing and Recording

Positional changes of the CI actuator were detected using a point laser sensor (IL-100 Intelligent Laser sensor, Keyence Corp., Japan). The displacement data were amplified using an IL-1000 amplifier unit (Keyence Corp., Japan). These data are transferred to the computer using a data acquisition board (USB-9239, National Instruments, United States) at a sampling frequency of 1000 Hz.

At the same time, the motion was captured through microscope cameras (Dino-Lite USB microscope cameras, Dunwell Tech Inc., United States). This series of photographs was analyzed through DinoCapture 2.0 (Dunwell Tech Inc., United States) and ImageJ software (National Institutes of Health, United States) to extract the motion trajectory.

## Results

### CI Actuator Configuration and Operation

Cell-inspired actuator is a resilient hemispherical actuator. At the hemispherical structure consisting of iron oxide–silicone mixture, there is carbon tape for conduction inside and a hole for air intake and exhaust (Figure 1E). At the flat bottom, there is the carbon tape covered with PET film. When a voltage potential difference is applied to the CI actuator, electrostatic attraction occurs between the hemispherical part and the bottom substrate (Figure 1F). At the same time, the air existing inside is escaped through the air hole. This phenomenon is analogous to the fact that the water inside the motor cell exits through the membrane (Figure 1B). On the other hand, when the voltage potential difference is disappeared, the CI actuator returns to its original shape by elasticity, and air flows into the CI actuator through the air hole (Figure 1G). The movement of air is essentially accompanied by the motion of the actuator and is similar to the way in which water flows back through the membrane as the motor cell recovers (Figure 1C).

### CI Actuator Movement

Cell-inspired actuator has volume change by electric input. Herein, we analyze the actuation performance of the CI actuator by size variation and input level change. First of all, we fabricated the CI actuators with various hemisphere radius (*r*) and height (*h*) and observed differences in motion.

We test the CI actuators with heights of 1.5, 2.5, and 3.5 mm at the same radius of 7 mm with a voltage difference of 5 kV. The displacement is 0.13 ± 0.03 mm when the height is 1.5 mm and 0.09 ± 0.01 mm when the height is 2.5 mm (Supplementary Table 1). The electrostatic attraction acts more strongly as the height became shorter. In addition, when the height of the CI actuator is more than 4 mm, the actuator does not move.

Additionally, we test another sample group with a radius of 14.5 mm with 5 kV input. Similarly, the samples have heights of 1.5, 2.5, and 3.5 mm. As a result, when *h* = 1.5 mm, the hemispherical part sticks to the bottom substrate, but it is sometimes difficult to return due to strong electrostatic attraction. When *h* = 2.5 mm, the actuator shows the greatest motion displacement with 0.27 ± 0.02 mm. When the height is 3.5 mm, it moves in the range of 0.15 ± 0.02 mm.

The forces mainly involved in the motion of the CI actuator can be considered as two categories: electrostatic attraction and elasticity. Electrostatic attraction is related to operate the actuator downward and elasticity is relevant to return to its original shape. In other words, electrostatic attraction plays a transformation role and elasticity plays a recovery role in the actuator motion.

Electrostatic attraction is directly influenced by the surface area and the distance between two electrodes. As the distance between two electrodes gets closer, the working force increases. Also, a larger surface area results in a larger force.

The CI actuator has a hemispherical shape, providing a larger surface area compared to two parallel plate structures. The surface area is proportional to the electrostatic attraction, which is an advantage in gaining force. In addition, since the gap between the two electrodes becomes narrower from the height of the peak, this also makes the actuator strong.

The CI actuator has equatorial radius larger than polar radius, so it is oblate hemispheroid (Tietze, 1965). It does not have two electrodes in parallel, but each point of the electrodes has different distances *z*_0_ + *z*. We comment that the origin of the *z*-axis is the top of PET film. Therefore, it is necessary to consider the electrostatic attraction for each segment. An oblate spheroid has a surface of revolution obtained by rotating an ellipse about its minor axis (Hilbert and Cohn-Vossen, 1999). Based on it, we obtain the following equation about the electrostatic attraction (*F*_*e*_) on a perfect oblate hemispheroid acting at the initial time. Electrostatic attraction acts between planes that are orthogonal to the oblate hemispheroid. Therefore, in consideration of this, we calculated the electro attraction force as orthorectified.

$$F_{e}=\varepsilon_{0}\,\pi \,r\,{\left(\Delta \,V\right)}^{2}\,\int_{0}^{h}\frac{\sqrt{h^{4}+\left(r-h\right)\,\left(r+h\right)\,z^{2}}}{h^{2}\,{\left(z_{0}+z\right)}^{2}}$$

$$\cos\left(\tan^{-1}\frac{h\,\sqrt{h^{2}-z^{2}}}{r\,z}\right)\,d\,z$$

where ε_0_, Δ*V*, and *z*_0_ are the electric permittivity of free space, the voltage difference, and a slight gap between the bottom substrate and lowest point in the CI actuator, respectively. As mentioned above, *r*, *h*, and *z* signify the hemisphere radius, height, and distance between two electrodes, respectively. The theoretical value and experimental setup value is stated in Supplementary Table 2. Also, the calculation values according to the size variation sample we made are presented in Supplementary Table 3.

**FIGURE 2:**
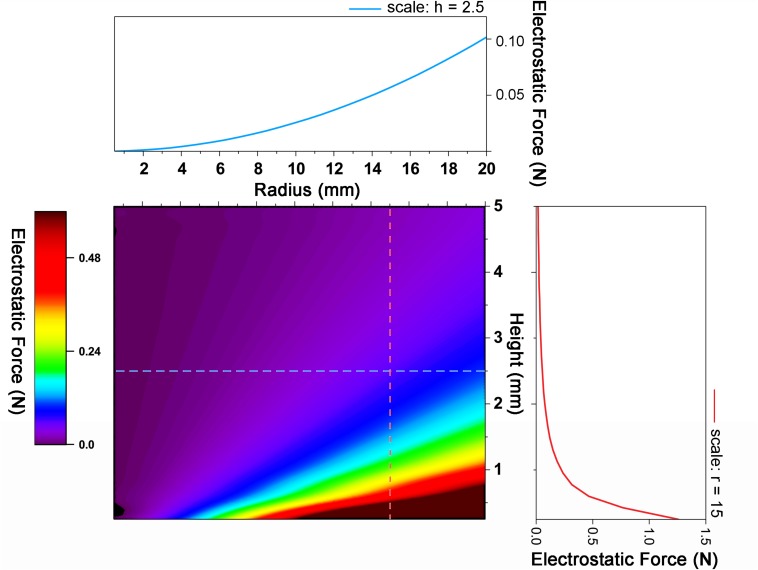
Graph of electrostatic force change with radius and height change. As the radius increases and the height decreases, the electrostatic force increases.

The actual displacement tendency corresponds to the electrostatic force. The largest electrostatic force was applied when *r* = 14.5 mm and *h* = 2.5 mm, and the smallest electrostatic force was applied when *r* = 7 mm and *h* = 2.5 mm. The electrostatic attraction when *r* = 14.5 mm and *h* = 2.5 mm is almost twice as large as *r* = 7 mm and *h* = 2.5 mm and the *r-*value is more dominant than the value of *h* (Figure 2). It is because the electrostatic force is inversely proportional to the distance to the substrate and is proportional to the radius. The closer to the bottom substrate and the larger the surface area, the greater is the electrostatic force; however, at too close distances, the movement of the actuator may be disturbed by interfering with recovery. Thus, according to the purpose, the distance to the substrate and the radius of the actuator should be determined. Figure 2 is the value to be based on this decision. The theoretical value shows that when *h* is less than 0.25 mm and *r* is greater than 18.5 mm, and an electrostatic force of 2 Nor more acts. Therefore, the numerical values of the following experiment are fixed with *r* = 14.5 mm and *h* = 2.5 mm.

The CI actuator has a resonant frequency of 21.7 Hz. The electrostatic energy is approximately 1.45 × 10^–5^ J. The mass for one CI actuator is 8.3 × 10^–4^ kg; thus, specific energy is 1.7 × 10^–2^J/kg. At the maximum input power of the high-voltage amplifier *P* = 1 W, the energy efficiency for 1 s is estimated as 1.45 × 10^–3^%.

**FIGURE 3:**
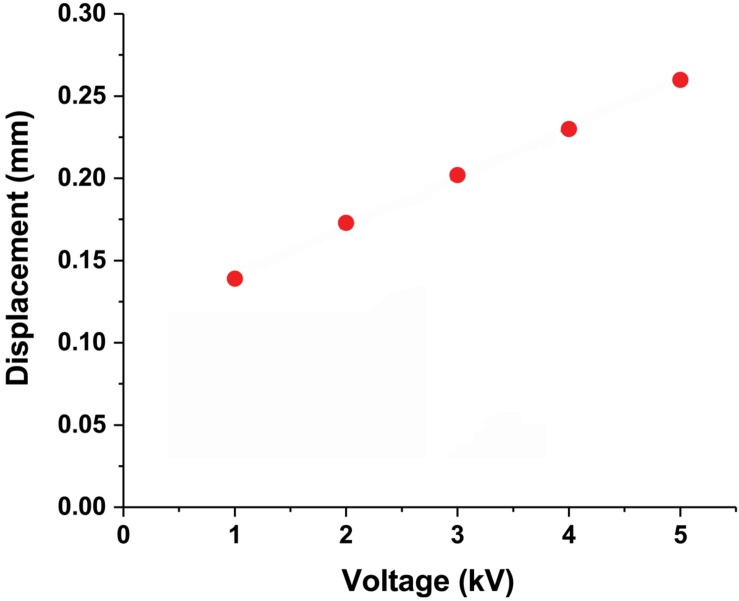
CI actuator displacement due to voltage difference. The CI actuator displacement changes proportionally when the voltage difference is from 1 to 5 kV.

On the other hand, the electrostatic force is also closely related to the voltage difference. The movement of the CI actuator changes with the magnitude of the voltage. According to Eq. 1, as the voltage difference increases, the electrostatic force increases by the square. Displacement does not increase by the square as the force, but displacement increases proportionally as the voltage increases (Figure 3). When the voltage is increased about 5 times, the displacement increases 1.87 times. This is probably related to the elasticity to be recovered.

Changes in these parameters occur while the actuator is in operation. As the actuator moves closer to the floor, the electrostatic force becomes stronger because the gap between the two electrodes narrows. On the other hand, as the actuator is deformed, the force to return to its original shape is counted, which increases the repulsive force. This is why the position moves slightly compared to when the actuator first reacted. Comparing the value of the speed, you can see which force is dominant under which state. It is also possible to estimate the minimum force value to overcome the static friction of the actuator. The electrostatic force under the condition that the displacement of the actuator falls below 100 μm is 1.25 × 10^–2^ N. This shows that in order for the actuator to move more than 100 μm, the force between the two electrodes must be greater than 1.25 × 10^–2^ N.

The elasticity of the CI actuators plays a role in helping recovery. That is, elasticity is used as an energy barrier during operation (Marthelot et al., 2017). The elastic energy of the perfect shell is related to a numerical coefficient that depends only on Poisson’s ratio. The silicone rubber used in the CI actuator is a hyperelastic material, so the energy barrier is large, which can be defined as a large force to be recovered to its original shape: properties from supplier material data sheets density of 1.07 × 10^–9^ g/cm^3^, Young’s modulus of 68.9 kPa, the tensile strength at 100% strain, and a Poisson’s ratio of 0.499 (Roche et al., 2014). On the other hand, the CI actuator shows 50 kPa, tensile strength at 100% strain and 570 kPa, compressive stress test at 50% strain. The detailed material test information is shown in Supplementary Tables 4, 5. Also, the strain and compressive stress test curves are shown in Supplementary Figure 2.

The bending stiffness of the shell is directly related to the shell thickness (Ventsel and Krauthammer, 2001). As the thickness increases, the bending stiffness of the shell increases. If the shell is very thin, the bending stiffness is low and the load is mostly resisted by the membrane force. As the shell thickness increases, the bending stiffness increases and more of the shell begins to resist the load through bending and membrane action. Therefore, a thin shell is not strong enough to return to its original shape, and the large thickness of the shell disturbs actuator movement. Therefore, the thickness of the CI actuator is fixed to 0.5 mm throughout the experiment.

### CI Actuator Layer Structure and Displacement

**FIGURE 4:**
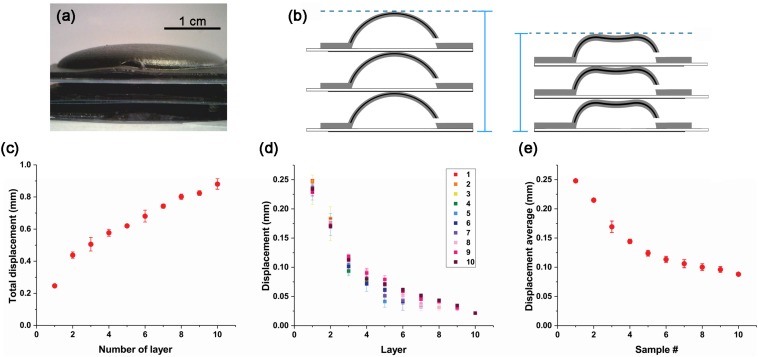
CI actuator layer displacement due to the number of layer. **(A)** CI actuator layer deposited in three layers. **(B)** CI actuator layer change schematic. When a voltage potential difference is applied, the CI actuators in each layer contract and the height changes. **(C)** Total displacement variation by number of layers. **(D)** Displacement variation of each layer from L_1_ to L_10_. **(E)** Average displacement variation of single layer from L_1_ to L_10_.

One of the biggest advantages of the CI actuator is that it can be assembled to create various shapes. In this study, we experimented to increase displacement by constructing the CI actuator stage (Figure 4A). We also studied the variation pattern of displacement. In order to balance this experiment, we made two actuators as one set and showed typical results, but various shapes are possible. Therefore, the schematic of Figure 4B is drawn by focusing on one CI actuator stack. When 5 kV is applied to the CI actuator layers, larger displacement can be obtained because each CI actuator that forms the stage shrinks or recovers at the same time (Figure 4B). The number of layers in a structure is indicated as L_#_: L_1_ for single layer, L_10_ for layer 10.

The displacement of each structure was investigated by increasing one layer at a time. In the case of a single layer (L_1_), it moved about 0.25 mm; however, when 10 layers are stacked (L_10_), the displacement is almost 0.9 mm. As the deformable gap increases, the total displacement increases (Figure 4C). However, this increase does not appear in proportion to the number of layers in the layer. When you see this trend, the displacement of more than 10 layers will gradually saturate. This is because the weight increases as the number of layer increases and the weight of the lower layer the CI actuator to lift is increased. Therefore, even if the number of layer increases, it has a limit displacement.

Movement characteristics can be seen more clearly by displacement of each layer (Figure 4D). For top layers that are not covered by other layers, the displacement is similar. But as the overlying layer increases, the displacement becomes smaller. When you see this tendency, if more layers are stacked, the displacement that each can move will be smaller. Therefore, as the number of layer increases, the displacement decreases inversely.

The average displacement can be obtained by dividing the displacement of the total structure by the number of layers constituting it (Figure 4E). In the case of L_10_, displacement average is smaller than 0.1 mm. This is only about one-third of the displacement that exists when it is independent.

The CI actuator single layer weighs approximately 4.54 g. That is, each time one layer increases, the force by weight increases by 4.54 × 10^–2^ N. It can be assumed that the bottom layer in L_10_ supports 4.01 × 10^–1^ N compared to the L_1_.

### Sensor Integrated CI Actuator

We aim at a motor CI system that can independently control the movement of each actuator even when multiple actuators are combined to form a complex structure. At that case, real-time sensing of each actuator motion is very important. It is easy to monitor a single actuator motion in a plane, but it is difficult to detect actuators’ motion in real-time when the structure is complicated. In order to detect the movement of the actuator that is not exposed to the outside, we can insert a piezo film sensor in the actuator to monitor the movement of the actuator in real-time. The CI actuator can be integrated with the vibration sensor (Supplementary Figure 3A). We also applied 5 kV to operate the actuator. The high-voltage could damage the sensor. To prevent this, a piezo film sensor was thinly covered with silicone and hardened together (Supplementary Figure 3B). A piezo film sensor was placed on the fabricated CI actuator and hardened with silicone. The point laser sensor and the piezo film sensor were operated at the same time to see if the sensor sensed the motion of the CI actuator accurately (Supplementary Figure 3C).

**FIGURE 5:**
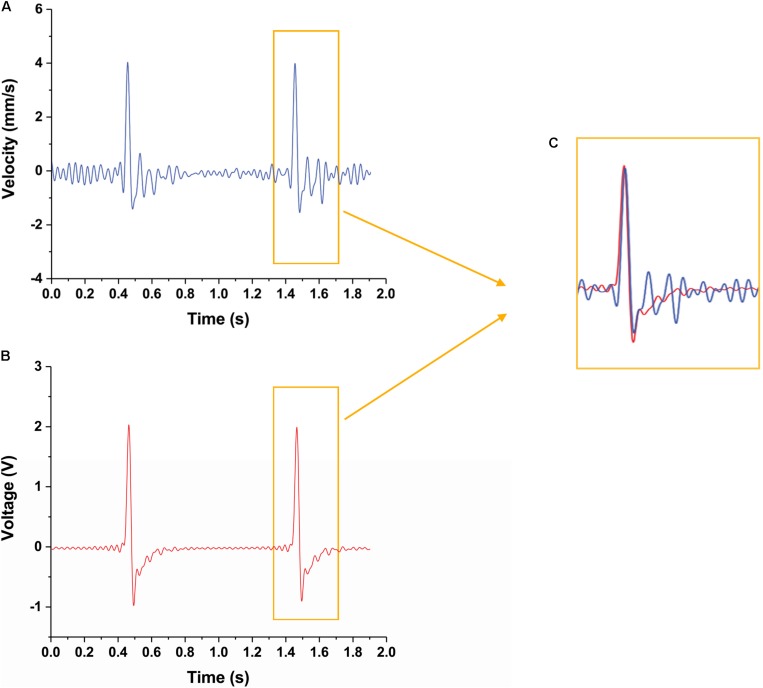
Velocity change of CI actuator detected by two different sensors. **(A)** Velocity change of the CI actuator detected by the laser sensor. **(B)** Velocity change of the CI actuator detected by the piezo sensor. **(C)** Peak magnification for comparing the detected velocity results in detail. It is shown that the velocity changes perceived by each sensor are similar to each other.

The velocities during the movement of the CI actuator are plotted in Figure 5. Since acquired data from the point laser sensor are the position changes of the CI actuator, the velocity was obtained by differentiating. The velocity measured by the point laser (Figure 5A) and the velocity measured by the vibration sensor (Figure 5B) showed consistent patterns of change (Figure 5C). Although there are some differences in the graphs, the two graphs are very similar given the following points: the noise value of the sensor, the point laser sensor value is the derivative of the displacement, and the vibration sensor value is the filtered point. Thus, it is possible to detect the movement of the CI actuator in real-time with the built-in sensor. Also, the CI actuator can be controlled in real-time by using this value.

## Discussion

In this study, a new type of CI actuator was developed and its performance was evaluated. The CI actuator proposed in this study is an actuator that utilizes electrostatic force and material characteristics. The CI actuators are very lightweight with flexible motion compared to conventional rigid actuators. It is operated by an electrostatic force and is recovered to the off state by the characteristics of the material. It is made of silicone and iron oxide, which is very cheap and easy to handle, and its size and shape can be freely modified. Also, the CI actuator proposed in this study tries to compensate for the two disadvantages of the existing soft actuator. First, actuators using air as a dielectric material are small and weak in displacement. To compensate for this, we proposed a multi-layer structure and succeeded in moving to the scale of millimeters. This will allow for greater displacement by adjusting the current applied to the actuator. It is also a big advantage to embed sensitive sensors in electrostatic actuators that utilize high voltages. We confirmed that the sensor works well without noise compared to the laser sensor. This has the advantage of detecting motion in real-time even if the actuator is positioned inside. The CI actuator has the advantage of being capable of motion detection with a built-in sensor without any additional equipment.

There are also many other strengths that can be highlighted. CI actuators have different forces to confront each other. If these characteristics are designed well for the purpose, it is possible to manufacture more effective actuators.

1. Size: CI actuators can be manufactured to any size. As the size of the CI actuator increases, the required force increases, but as the size of the electrostatic force increases, it is possible to procure the necessary force.
2. Material concentration and structure optimization: CI actuators have the advantage of using electrostatic attraction, and recovery can be done quickly due to their own elasticity. Due to the elasticity of the material itself, silicone can act as an obstacle to on state movement, but it has the advantage of not requiring any other force during recovery. The elastic force can be controlled by varying the thickness or composition ratio of the structure.
3. Supporting force for multiplex structure: CI actuators have the advantage of detecting motion in real-time and enabling independent movement. However, in order to support the multiplex structure, it is possible to increase the tension of the structure itself so that the CI actuator can better support the structure.

These various strengths will be key to enabling CI actuators to be used in more applications.

Despite these advantages, there are also some concerns. Given the possibility that the CI actuator can move at very high speeds, the size of the air hole can have a big impact on the movement. When the radius of the hole is half, the surface area decreases by a quarter and the pressure increases accordingly. Therefore, if we want to manufacture a high-speed actuator, we will need a suitable air hole size accordingly.

## Conclusion

In this study, a CI actuator was fabricated and tested with a layer structure form. The CI actuator is able to move about 0.2 mm, and the height variation is larger when the CI actuator has a layered structure. In addition, this actuator incorporates a sensor to monitor movement in real-time.

Since the actuator is inspired by the cell, the body can move independently, and it is also possible to control the movement through various arrangements. This movement can also be detected and reported in real-time through the sensor. Therefore, we will make use of this actuator to realize a stimuli-responsive actuator that exhibits a large range of motion including bending and twisting. In the future, the actuator can be used as a gripper or a soft robot.

## Data Availability Statement

All datasets generated for this study are included in the article/Supplementary Material.

## Author Contributions

KS and YC planned and designed the experiments, analyzed the data, and wrote the manuscript. KS performed the fabrication and experiments.

## Conflict of Interest

The authors declare that the research was conducted in the absence of any commercial or financial relationships that could be construed as a potential conflict of interest.
