# Supplementary material for: Hemispherical Cell-Inspired Soft Actuator
Source: Front Bioeng Biotechnol. 2020 Feb 19;8:20. doi: 10.3389/fbioe.2020.00020 (PMC7042176; doi:10.3389/fbioe.2020.00020)
Supplement: Supplementary file 1 [file Table_1.DOCX]

Supplementary Material

# Supplementary Figures


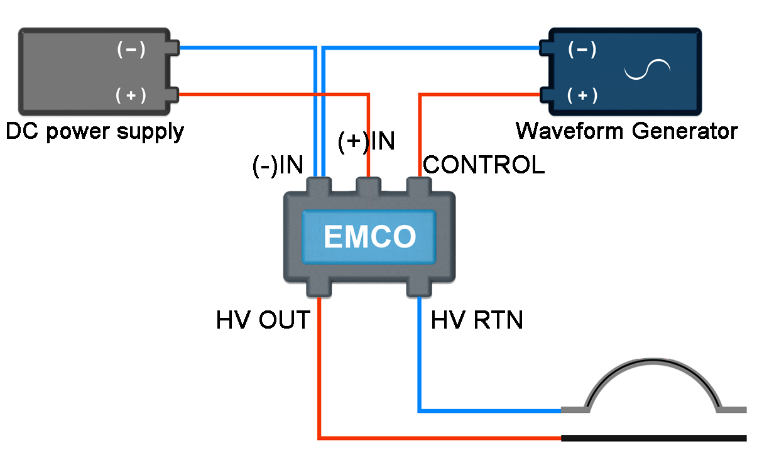


**Supplementary Figure 1.** **Schematic for circuit configuration and setup.** A power supply and a function generator were connected to the EMCO to supply high voltage to the actuator.

**
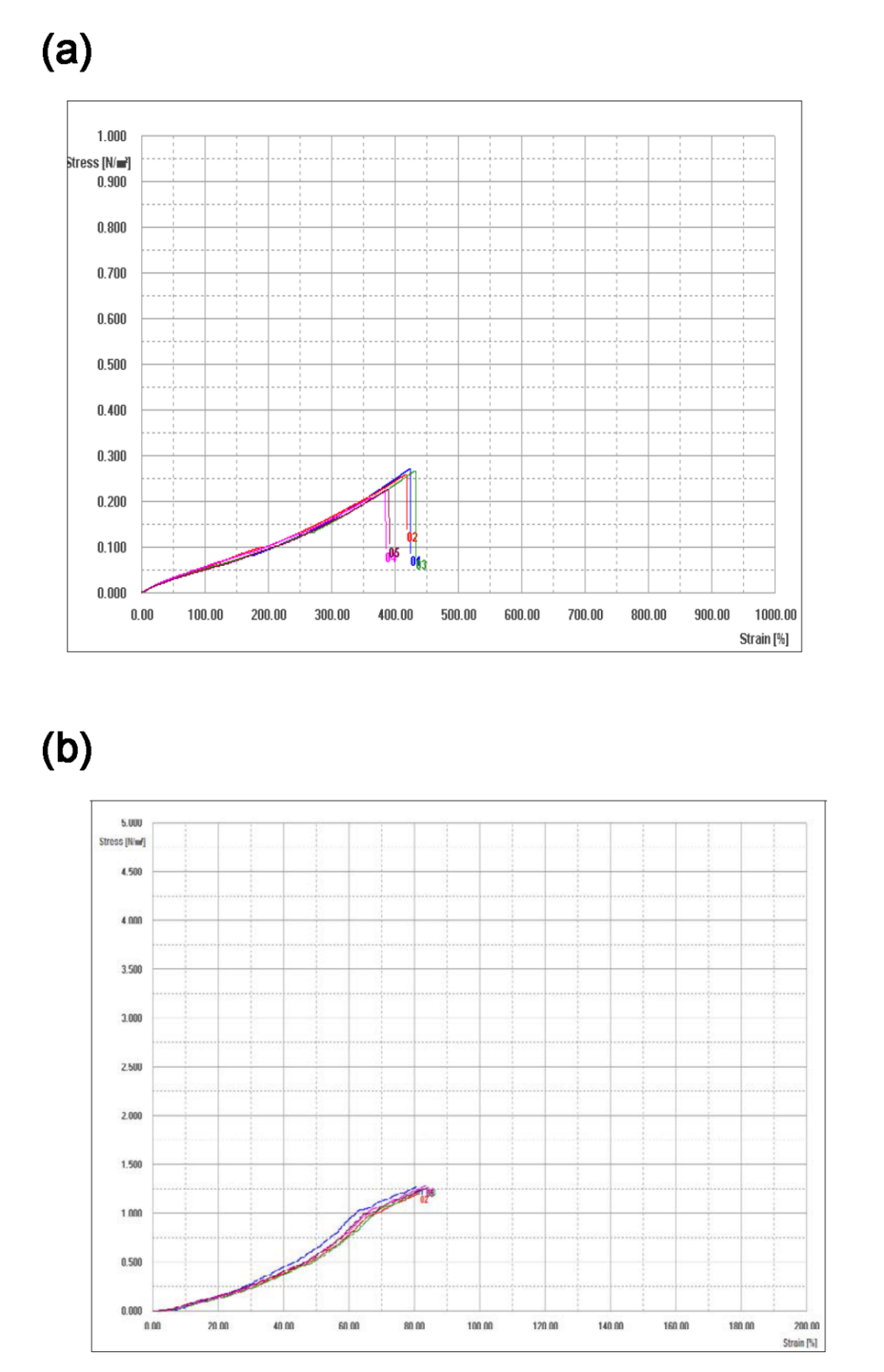
**

**Supplementary Figure 2.** **Material characteristic curve** (a) Tensile strength test. (b)compressive stress Test.


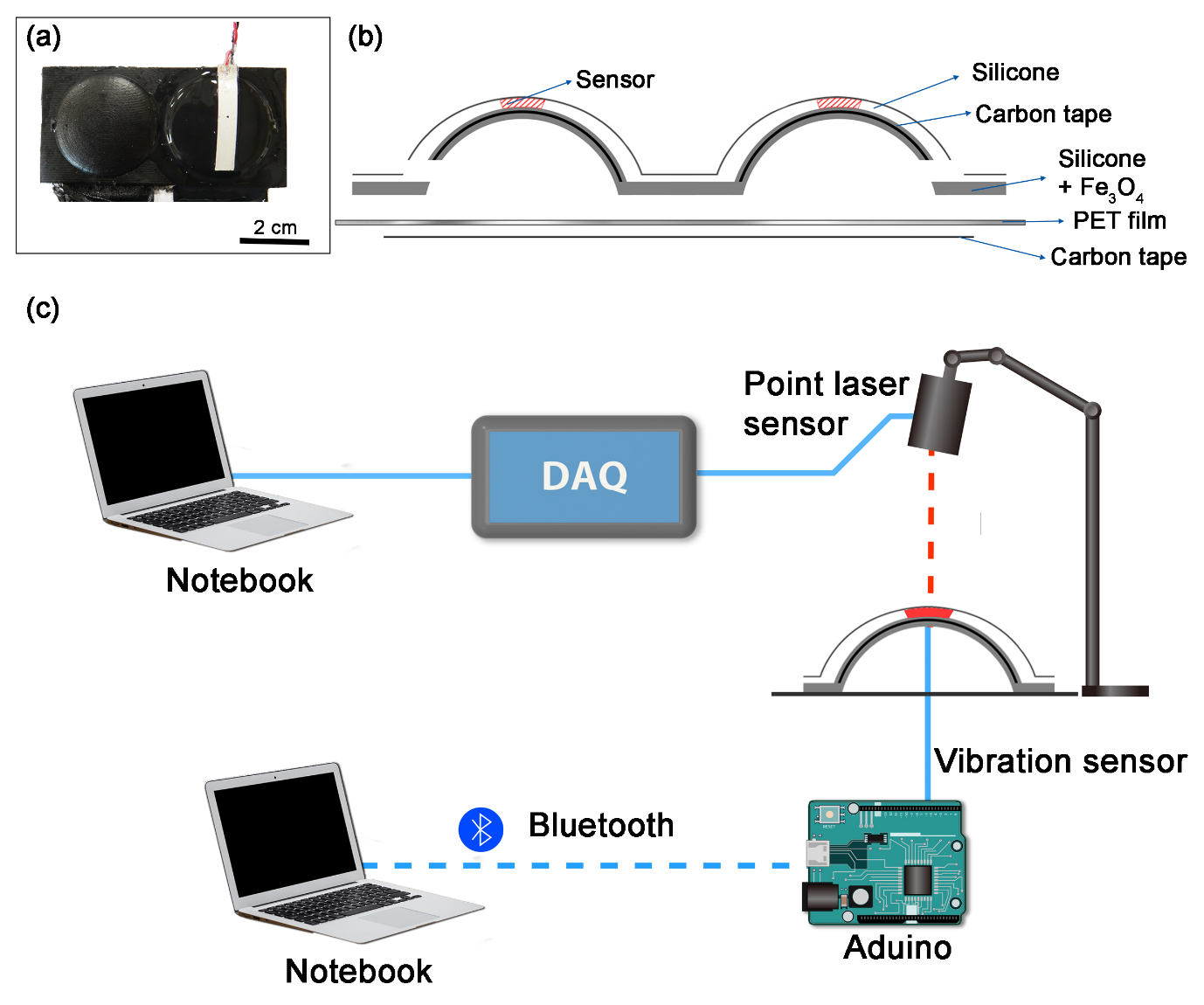


**Supplementary Figure 3.** **Piezoelectric Sensor Integrated CI actuator and Experimental set-up.** (a) Piezo-sensor integrated CI actuator. (b) Component schematic of a piezo-sensor integrated CI actuator. (c) Configuration for piezo sensor detection and point laser detection schematic.

# Supplementary Tables

**Supplementary Table 1. Motion change due to size variation**

|  | **Displacement average (mm)** | **Standard deviation** |
| --- | --- | --- |
| ***r* = 7 mm, *h* = 1.5 mm** | 0.13 | 0.03 |
| ***r* = 7 mm, *h* = 2.5 mm** | 0.09 | 0.01 |
| ***r* = 14.5 mm, *h* = 2.5 mm** | 0.27 | 0.02 |
| ***r* = 14.5 mm, *h* = 3.5 mm** | 0.15 | 0.02 |

* Sample number n = 4

**Supplementary Table 2. Experimental data and theoretical value for calculation**

| **Symbol** | **Definition** | **Value** |
| --- | --- | --- |
| *ε_0_* | Electric permittivity of free space | 8.85ⅹ10^-12^ F/m |
| *ΔV* | Voltage difference | 5 kV |
| *z_0_* | Gap between the bottom and lowest point in CI actuator | 0.1ⅹ10^-3^ m |

**Supplementary Table 3. Theoretical value due to size variation**

|  | **Electrostatic force** |
| --- | --- |
| ***r* = 7 mm, *h* = 1.5 mm** | 2.78 ⅹ 10^-2^ N |
| ***r* = 7 mm, *h* = 2.5 mm** | 1.25 ⅹ 10^-2^ N |
| ***r* = 14.5 mm, *h* = 2.5 mm** | 5.37 ⅹ 10^-2^ N |
| ***r* = 14.5 mm, *h* = 3.5 mm** | - 1. ⅹ 10^-2^N |

**Supplementary table 4. Material (Fe_3_O_4_ + silicone) tensile strength test**

| **Contents** | **Results** |
| --- | --- |
| **Tensile strength (N/mm^2^)** | 0.25 |
| **Tensile stress test @ 10% strain (N/mm^2^)** | 0.01 |
| **Tensile stress test @ 50% strain (N/mm^2^)** | 0.03 |
| **Tensile stress test @ 100% strain (N/mm^2^)** | 0.05 |

*Test velocity: 500 mm/min

**Supplementary table 5. Material (Fe_3_O_4_ + silicone) compressive stress Test**

| **Contents** | **Results** |
| --- | --- |
| **Compressive stress test @ 10% strain (N/mm^2^)** | 0.04 |
| **Compressive stress test @ 30% strain (N/mm^2^)** | 0.25 |
| **Compressive stress test @ 50% strain (N/mm^2^)** | 0.57 |

*Test velocity: 1.3 mm/min
